# Development and evaluation of a head-controlled wheelchair system for users with severe motor impairments

**DOI:** 10.1016/j.mex.2025.103485

**Published:** 2025-07-05

**Authors:** Abdelhakim Haddoun, Dâlel Djabri, Mallak Saidani, Mohamed Benbouzid

**Affiliations:** a University of Oum El Bouaghi, Institute of Technology, Oum El Bouaghi, Algeria; b Dalian University of Technology, School of Software Engineering, PR China; c University of Oum El Bouaghi, Faculty of Exact Sciences and Natural and Life Sciences, Algeria; d University of Brest, UMR CNRS 6027 IRDL, Brest, France

**Keywords:** Electric wheelchair, Mobility impairment, Autonomy, Gesture control, Occipital control, Innovative control systems, Assisted mobility

## Abstract

This paper presents an innovative and accessible hands-free wheelchair control system designed for individuals with severe motor impairments, particularly tetraplegic users. Unlike traditional joystick-based systems, which are often unsuitable for users with quadriplegia, our system relies on intuitive head-motion detection to enhance autonomy and ease of use. The system consists of a wearable motion-sensing cap equipped with an MPU-6050 sensor which is a 6-axis Inertial Measurement Unit (IMU) to capture head gestures, processed by an ATmega328 microcontroller (a low-power 8-bit AVR microcontroller widely used in embedded systems) integrated on an Arduino Nano development board. Wireless commands are transmitted via a Bluetooth module (HC-05) to the wheelchair’s control unit consisting of an Arduino Uno microcontroller and BTS7960 motor drivers — high-power H-bridge modules that enable bidirectional control of DC motors. The operational flow, including signal processing, gesture interpretation, and wireless transmission, is structured following a detailed flowchart-based design. Experimental results indicate a high response rate and directional accuracy of over 90 % using a 45° head tilt. The optimal safe speed was determined to be 1.87 km/h with a Pulse Width Modulation (PWM) value of 180. Rather than designing a mechanical chassis from scratch, a commercially available electric wheelchair was modified by removing its joystick interface, allowing seamless integration of the head-controlled system. These findings validate the system’s usability and precision under real-world conditions. By eliminating manual input and emphasizing simplicity, the proposed solution holds strong potential as a scalable and low-cost mobility aid, especially in low-resource environments.

## Graphical abstract

**Figure:**
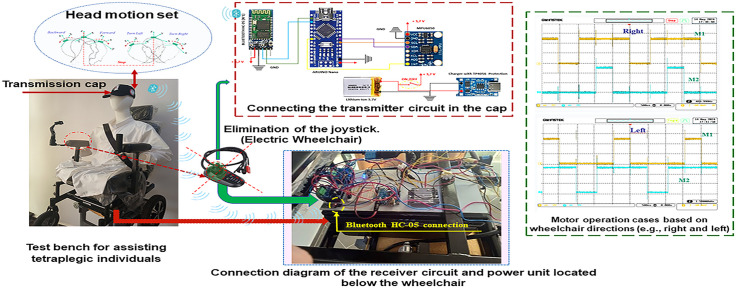

## Related research article

None.

• A head-motion-based control system using an MPU-6050 sensor and Arduino-based processing.
• Wireless Bluetooth communication ensures seamless transmission to the wheelchair control unit.
• Experimental validation confirms smooth and precise navigation for users with severe disabilities.

**Specifications table**

**Table:** 

**Subject area**	Engineering
**More specific subject area**	Assistive Technology, Human-Machine Interaction, Wheelchair Control. Systems
**Name of your method**	Head-Gesture-Based Control System for Electric Wheelchairs.
**Name and reference of original method**	Not applicable.
**Resource availability**	Custom-built hardware (MPU6050, HC-05, Arduino Mega, BTS7960), Open-source software (Arduino IDE, Bluetooth communication protocols), Dataset for training/testing head gesture recognition.

## Background

Mobility impairment remains a significant challenge for individuals with severe motor disabilities, profoundly affecting independence and quality of life. Traditional electric wheelchair control methods, such as joystick-based interfaces, are often inaccessible for users with conditions like quadriplegia or severe neuromuscular disorders. In recent years, alternative input systems such as voice commands, eye tracking, sip-and-puff devices, and brain-computer interfaces (BCIs) have been developed to overcome these limitations [[1], [2], [3]]. However, these solutions often involve high implementation costs, complex calibration procedures, or require significant cognitive or physical effort, limiting their usability and scalability in low-resource settings [4,5].

To overcome these barriers, this work introduces an intuitive and scalable head-controlled electric wheelchair system based on inertial sensing. The approach relies on the MPU-6050 sensor, a 6-axis Inertial Measurement Unit (IMU), which captures head movements and converts them into directional commands. These signals are processed in real time by an ATmega328 microcontroller (Arduino Nano) and transmitted wirelessly using a Bluetooth HC-05 module to the receiver unit. The wheelchair’s control unit, based on an Arduino Uno board, interprets the received signals and manages the motors using BTS7960 H-bridge drivers. This design enables a contactless and low-effort control strategy suitable for users with limited motor abilities.

The navigation process is further optimized by calibrating angular thresholds based on MPU-6050 data. Although head movements may exceed 45° visually, the system detects direction commands using pre-programmed thresholds around ±45°, ensuring consistent directional control without requiring fine motor precision or complex coordination [6].

Compared to BCI-based or vision-based systems, the present method emphasizes simplicity, affordability, and integration feasibility. No mechanical redesign is required, as the joystick interface is removed and replaced by the custom control module. This reduces both cost and technical barriers to implementation.

Several previous studies have proposed head-movement control systems for mobility assistance [[7], [8], [9]], but many of them are either limited to laboratory testing or depend on specialized hardware. This contribution addresses a critical gap by offering a reproducible, open-source alternative validated under real-world conditions.

The primary objective of this study is to design and experimentally validate a modular, gesture-based control system for electric wheelchairs that prioritizes accessibility, affordability, and usability. Specifically, this work offers three key contributions: (1) the development of a low-cost, modular behavioral command interface for electric wheelchairs; (2) the integration of head movement signals with real-time motor control; and (3) the validation of the system in a representative environment. Compared to existing high-cost commercial solutions, our system offers improved affordability, local reproducibility, and intuitive interaction adapted to users with severe motor disabilities.

The novelty of this work lies in the combination of behavioral signal processing (head movements) with an open-source embedded system for wheelchair control, designed specifically for patients with severe disabilities in resource-limited settings. Unlike commercial electroencephalography (EEG) or eye-tracking systems, our approach prioritizes low-cost components, ease of local assembly, and adaptability, making it both inclusive and scalable for real-world application.

## Method details

The proposed system was implemented using a head-mounted motion-sensing module consisting of an MPU-6050 IMU embedded in a lightweight cap. Head gestures were captured in real time and processed by an ATmega328 microcontroller on an Arduino Nano board. Four directional commands (forward, backward, left, right) were extracted from the IMU data based on angular thresholds calibrated around ±45°. These commands were transmitted wirelessly via a Bluetooth HC-05 module to the receiver unit, which consists of an Arduino Uno and two BTS7960 motor drivers connected to the wheelchair’s motors. The system was designed to be non-invasive, low-cost, and adaptable to standard commercial electric wheelchairs.

This solution was developed with a strong focus on simplicity, cost-efficiency, and accessibility, enabling easy replication using off-the-shelf electronic components [8,9].

To address the specific needs of individuals with motor impairments who cannot operate conventional joystick-based controls, the present approach leverages intuitive head movements for real-time mobility control [6,11].

The increasing demand for electric wheelchairs, driven by factors such as congenital disabilities, accidents, and aging, has led to the need for more accessible and intuitive alternatives [2,12].

**Fig. 1:**
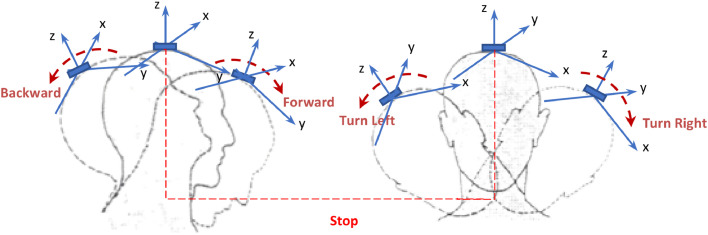
Series of movements to control the electric wheelchair.

The system described here primarily utilizes an MPU-6050 module, which integrates accelerometers and gyroscopes to detect tilts and rotations of the user’s head, as shown in Fig. 1. Similar IMU-based head control approaches have been explored in previous studies, where the implementation of a fuzzy logic controller has demonstrated improvements in the precision of wheelchair movements [11]. This figure illustrates a series of head movements used to control the wheelchair, translating the user’s natural gestures into precise and fluid commands. Head gesture-based control has been studied in multiple research efforts to provide a hands-free and efficient navigation method for individuals with severe motor impairments [13,14]. Moreover, assistive robotic exoskeletons for head and neck rehabilitation have contributed to improving motion tracking and user comfort in mobility applications [5].

The MPU6050 is a sophisticated motion sensor capable of precisely measuring both linear accelerations and angular rotations. When attached to a helmet worn by the user, it tracks head movements in real-time, ensuring immediate responsiveness and improving the user’s ability to navigate their environment with ease [15,16].

The data collected by the MPU6050 motion sensor is continuously monitored and transmitted to an ATMega328 microcontroller (Arduino Nano) for further processing. This microcontroller acts as the brain of the control system, where it interprets the head movement data captured by the MPU6050 sensor and converts it into actionable commands for controlling the wheelchair [17]. These commands determine the wheelchair's steering and speed based on the user’s head movements [10].

To ensure seamless and efficient communication, the processed data is wirelessly transmitted to the wheelchair's motor control system via a Bluetooth HC-05 module (No.1). This Bluetooth module allows for a reliable and responsive connection between the wearable device and the wheelchair, enabling real-time control without the need for direct physical contact.

**Fig. 2:**
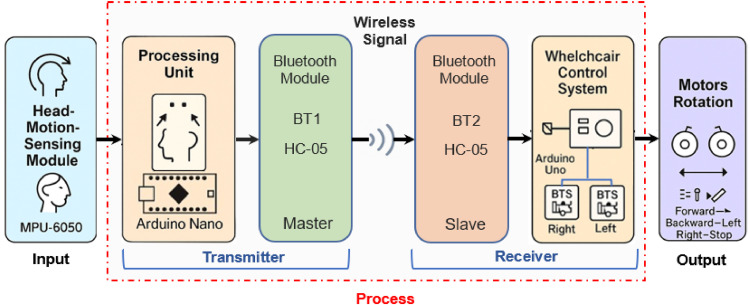
Block diagram of the head-motion-based electric wheelchair control system.

An overview of the full control loop is presented in Fig. 2, which illustrates the system architecture, from gesture acquisition to motor activation.

**Fig. 3:**
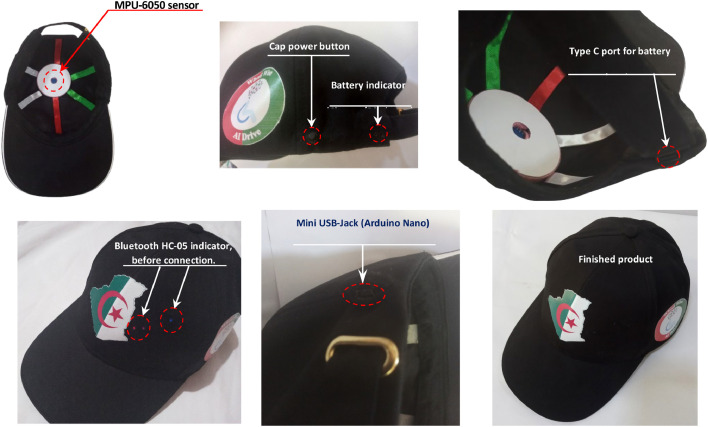
The transmitter cap.

Fig. 3 shows the transmitter cap worn by the user, designed to detect head movements and wirelessly transmit control commands to the electric wheelchair. The system incorporates an MPU-6050 motion sensor, mounted on the top of the cap, to capture head accelerations and rotations. On the side of the cap, a power button and a battery level indicator allow the user to easily manage the system’s power status. A built-in Type-C charging port, located inside the cap, enables convenient recharging of the onboard battery.

The cap houses an Arduino Nano board, accessible via a Mini USB jack, which processes sensor data in real-time. A Bluetooth HC-05 module, with a visible LED indicator on the front, ensures wireless communication with the receiver unit attached to the wheelchair. The compact and ergonomic arrangement of components ensures user comfort while minimizing bulk. The final product image illustrates the neat and discreet integration of electronic components into a standard cap, offering a wearable, user-friendly, and aesthetically pleasing design.

**Fig. 4:**
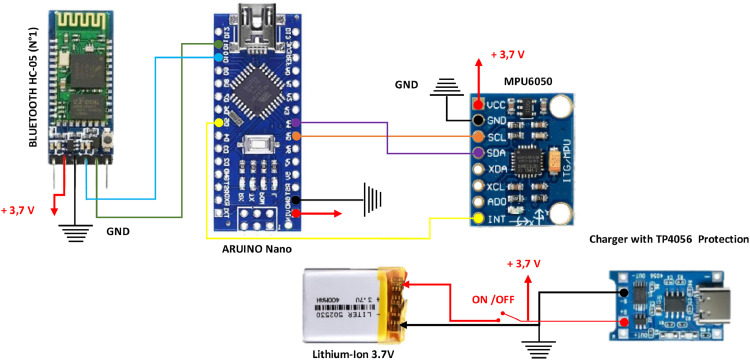
Connecting the transmitter circuit in the cap.

Finally, Fig. 4 provides a detailed view of the electronic components integrated into the wearable cap. It shows the layout of the MPU6050 sensor, microcontroller, and Bluetooth module, along with their interconnections. This arrangement ensures the system operates optimally, with the components arranged in a compact, user-friendly design that maximizes performance while minimizing physical strain for the user.

The captured data is sent to an Arduino Nano where it is processed to generate commands for the wheelchair motors. The microcontroller interprets the head movements in real-time and converts them into motor control signals, ensuring smooth, intuitive, and responsive operation.

The receiver module comprises essential components for wheelchair control, including an Arduino Uno, a Bluetooth HC-05 module (No.2), two BTS7960 motor drivers, and a Sochi DC 24 V voltage stabilizer (10–36 V to 24 V).

**Fig. 5:**
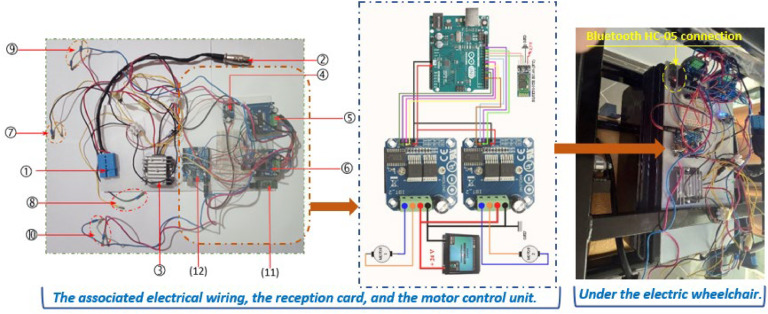
Connection diagram of the receiver circuit and power unit.

The voltage stabilizer ensures separation of the power supply to the motor drivers from that of the electromagnetic brakes and Arduino Uno board. The Arduino Uno receives commands via Bluetooth and controls the motors accordingly, ensuring precise and dynamic responses. To ensure smooth and reliable operation, the Arduino Uno is powered by a DC-DC boost step-up power module, which converts the input voltage (5V-35 V) to a stable 12 V output. The components are securely mounted on a 34.50 × 18 cm acrylic plate placed beneath the wheelchair for discreet integration, as shown in Fig. 5. The layout of these components is designed to optimize space while maintaining proper ventilation. Table 1 details the receiver module and motor control components.

**Table 1:** Receiver module and motor control components.

N°	Components
1	24 V battery plug
2	Plug for battery charger
3	Sochi DC 24 V voltage stabilizer, 10–36 V to 24V
4	DC-DC boost step-up power module output 5V-35V
5	Driver BTS7960 - Straight
6	Driver BTS7960 - Left
7	To electromagnetic brake (right-hand motor)
8	To electromagnetic brake (left-hand motor)
9	To Right motor
10	To left motor
11	Bluetooth HC-05 (No. 2)
12	Arduino Uno

This configuration provides a functional, high-performance alternative to traditional joystick control for electric wheelchairs. The integration of wireless communication via Bluetooth and motorized control components ensures the system is intuitive, ergonomic, and highly effective. The key benefits of this control system include improved accessibility, enabling users with hand or arm limitations to control the wheelchair more easily; increased comfort, eliminating the need for joystick manipulation and reducing physical strain; reactivity and precision, offering more intuitive and precise control through natural head movements; easy integration, making the system compatible with most existing electric wheelchairs; customization, allowing adjustments to meet the specific needs of the user; improved safety, minimizing the risk of false manipulations and improving response to user movements; and increased autonomy, enhancing users' freedom and independence, making daily activities more manageable.

**Fig. 6:**
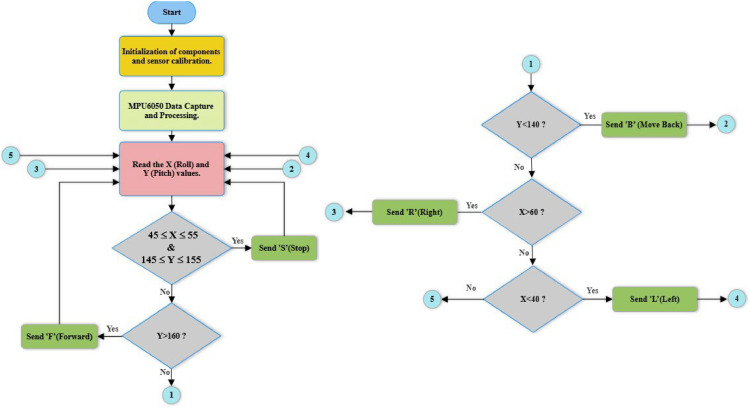
Flowchart of the transmitter module.

The flowchart in Fig. 6 illustrates the operational process of the transmitter module of the electric wheelchair gesture control system, which focuses on the MPU6050 module and an Arduino microcontroller. The program, compiled and uploaded via the Arduino IDE, captures the user’s head movements, interprets them, and converts them into control commands for the wheelchair. It begins with the initialization of components and the calibration of sensors, ensuring accurate movement measurement and responsiveness. Real-time data from the MPU6050′s integrated accelerometers and gyroscopes is continuously captured and processed, forming the foundation for precise steering and speed control.

The system’s functionality revolves around calculating the values of X (roll) and Y (pitch), which define the direction of movement. Roll represents the inclination of the sensor around the longitudinal axis (left-right), while pitch represents the inclination around the lateral axis (forward-backward). These raw values are remapped using the map function to ensure proper scaling and interpretation. Roll is limited to a range of −100 to 100 degrees and converted into an X value between 0 (extreme left) and 100 (extreme right). Similarly, pitch is constrained to −100 to 100 degrees and mapped into a Y value between 100 (extreme backward) and 200 (extreme forward).

Once the X and Y values are calculated, the system interprets them to determine the wheelchair’s movement direction. Specific thresholds are established to define different zones of motion:

• Neutral position (Stop): If 45≤*X* ≤ 55 and 145≤*Y* ≤ 155, the system identifies a neutral zone, where no significant tilt is detected, and the wheelchair remains stationary. The command 'S' is sent to the receiver.
• Forward motion: If *Y* > 160, the user tilts the sensor forward, generating a 'F' (Forward) command, causing the wheelchair to move ahead.
• Backward motion: If *Y* < 140, a backward tilt is detected, resulting in the command 'B' (Backward) for reversing.
• Right turn: If *X* > 60, the system identifies a tilt to the right and sends the command 'R' (Right) to turn the wheelchair in that direction.
• Left turn: If *X* < 40, a leftward tilt is recognized, and the command 'L' (Left) is issued for leftward movement.

The mapping and interpretation of X and Y ensure smooth and intuitive wheelchair operation. X primarily controls left-right movement, while Y governs forward-backward motion. The neutral zone (X between 45 and 55, and Y between 145 and 155) is critical for stability, preventing unintentional movements when the sensor is held steady. The sensitivity and precision of these ranges are carefully chosen to capture deliberate head gestures while filtering out minor oscillations or noise.

This approach enables safe, fluid, and user-friendly control of the wheelchair. Each step in the workflow, from sensor calibration to command transmission, is essential for delivering reliable and responsive navigation, as highlighted in the flowchart.

**Fig. 7:**
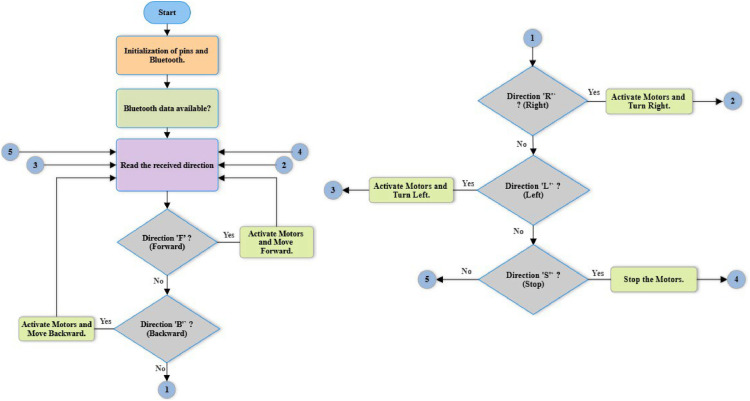
Flowchart of the receiver module.

The flowchart in Fig. 7 illustrates the control process of the receiver module of an electric wheelchair, which operates via Bluetooth communication and receives commands from the transmitter cap. The system employs the HC-05 Bluetooth module for wireless data transmission and controls the motors using PWM (Pulse Width Modulation) signals. At initialization, the necessary pins for motor control are configured as outputs, and all motors are stopped to ensure a safe startup. The system then enters a continuous loop where it monitors incoming data via the Bluetooth connection.

When a command is received from the transmitter cap, it is interpreted and executed accordingly. Receiving the letter ‘F’ activates forward movement, while ‘B’ initiates reverse motion. Commands ‘R’ and ‘L’ allow turning right and left, respectively. The ‘S’ command immediately stops all motor activity. These actions are performed by adjusting the speed and direction of the motors through PWM signals applied to the corresponding control pins. Each time a command is processed, the system returns to the "Read Received Direction" block, ensuring that it remains responsive to new inputs.

Before executing any movement, the motor activation function is called to power the system. Conversely, when stopping the wheelchair, the power supply to the motors is completely deactivated to prevent unwanted motion. Forward and reverse movements are achieved by simultaneously activating the left and right motors in the same or opposite directions with controlled intensity. Turning is managed by adjusting the speed of one motor relative to the other, enabling precise and smooth rotations. Stopping is ensured by setting all PWM signals to zero and cutting off motor power, preventing unnecessary energy consumption. This control system guarantees smooth, precise, and responsive wheelchair operation. By continuously reading Bluetooth commands and dynamically adjusting motor parameters, it enhances maneuverability, user comfort, and overall control reliability.

## Results

This section presents a structured analysis of the experimental results obtained from the developed prototype. We begin by detailing the relationship between the pulse width modulation (PWM) signal and the wheelchair's translational speed, followed by an examination of motor behavior and real-time voltage responses. Subsequently, the overall method is validated through performance tests and user interaction analysis, including visual feedback.

### Pulse width determination and wheelchair speed

To evaluate the movement of the wheelchair, tests were conducted without any load over a distance of up to 6 m By varying the pulse width, the travel time was measured for each configuration. The results, summarized in Table 2, indicate that a maximum pulse width of 250 is required to achieve the wheelchair's maximum speed. Additionally, to determine the safe operating speed of the wheelchair, the measurements in Table 2 reveal a speed variation ranging from 1 to 2.6 km/h. Based on these findings, a pulse width of 180 is recommended, corresponding to a speed of 1.87 km/h. This configuration balances performance with user safety, ensuring the wheelchair operates smoothly and predictably.

**Table 2:** Wheelchair speed according to pulse width.

PWM Command (analog Write)	Driver output voltage (V)	Speed	
		**m/s**	**Km/h**
**50**	**2.3**	**0.107**	**0.38**
**100**	**9.23**	**0.28**	**1.00**
**180**	**16.61**	**0.514**	**1.85**
**200**	**18.53**	**0.573**	**2.06**
**225**	**20.90**	**0.651**	**2.34**
**250**	**23.20**	**0.726**	**2.61**

To ensure precise and proportional control of the wheelchair's movement, we implemented speed regulation via Pulse Width Modulation (PWM). The PWM signal controls the average voltage supplied to the DC motors, thereby modulating their rotational speed and, consequently, the translational speed of the wheelchair. The duty cycle *D* of the PWM signal, expressed as a percentage, directly influences the motor voltage *V*_motor_, which in turn affects the wheel's angular velocity ω Assuming linear motor behavior in the operational range, the following relationship can be established:

(1) $$V_{motor}=D\times V_{\sup ply}$$

(2) $$\omega =k_{1}\times V_{motor}=k_{1}\times D\times V_{\sup ply}$$

(3) v=r×ω=k×D

where:

• *V*_supply_ is the motor power supply voltage,
• *D* is the PWM duty cycle (0 ≤ *D* ≤ 1),
• ω is the angular velocity of the motor (rad/s),
• *r* is the radius of the wheel (m),
• *v* is the linear speed of the wheelchair (m/s),
• *k*_1_ and *k* = *k*_2_×*r*, are constants dependent on the motor characteristics and mechanical configuration.

These equations demonstrate that the linear speed of the wheelchair is directly proportional to the PWM duty cycle. This proportionality simplifies the mapping between user-intended gestures (via head movement) and corresponding speed commands.

This calibration allows us to define a safe and effective range for motor commands:

• Low-speed zone (e.g., 0.05–0.1 m/s) for precise maneuvers,
• Moderate speed zone (e.g., 0.1–0.18 m/s) for regular driving,
• High-speed zone (e.g., >0.18 m/s) for forward motion on open paths.

### Motor operation and signal analysis

The coordination and responsiveness of the motors are crucial for the wheelchair’s performance. Using an oscilloscope, voltage curves as a function of time were obtained, showing the command signals sent to the two motor drivers. These signals provide critical insights into the system’s real-time behavior. The precise visualization of these voltage variations enables fine-tuning of the control signals, ensuring smooth motor operation.

**Fig. 8:**
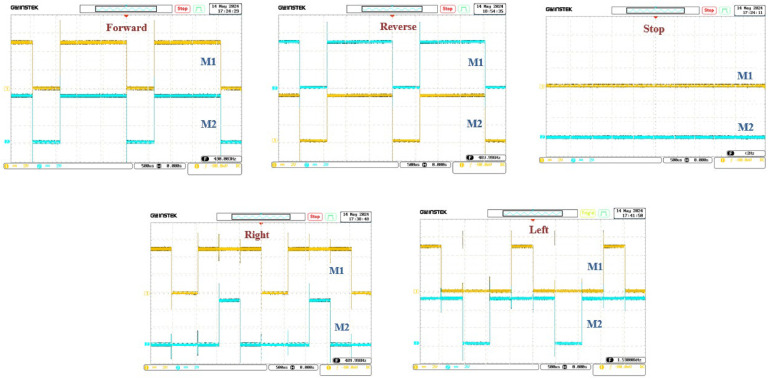
Motor operating cases according wheelchair directions.

For instance, as depicted in Fig. 8, when the x-axis tilt is <45°, and the y-axis value remains stable, both left and right motors rotate clockwise, causing the wheelchair to move forward. This synchronization ensures stable and reliable forward movement, enhancing the user experience and providing a safe, controlled motion. The motor control logic, detailed in Table 3, outlines the direction of rotation for each command, such as forward, backward, or turning, and is consistent with the observed motor behavior.

**Table 3:** Motor control logic.

Action	Right Front Motor	Right Rear Motor	Left Front Motor	Left Rear Motor	Description
Forward	LOW	HIGH	LOW	HIGH	**Both motors move forward.**
Backward	HIGH	LOW	HIGH	LOW	**Both motors move backward.**
Left	LOW	HIGH	HIGH	LOW	**Right motor moves faster than left.**
Right	HIGH	LOW	LOW	HIGH	**Left motor moves faster than right.**
Stop	LOW	LOW	LOW	LOW	**Motors are stopped and deactivated.**

The system continuously monitors incoming Bluetooth data, interpreting the received commands to determine the necessary motor actions. Forward movement, for instance, is achieved by rotating both motors in the same direction, while turning adjustments are made by varying the speed of one motor relative to the other. For backward motion, the motors are reversed, and stopping the wheelchair involves deactivating the PWM signals, effectively halting the motors.

Voltage measurements taken during operation reveal how each motor responds to these commands. The analysis of these signals ensures that the system remains responsive to user inputs, with each movement being performed smoothly and reliably. By observing these real-time voltage variations, adjustments can be made to optimize the performance of the motors, guaranteeing smooth transitions between commands and enhancing the overall user experience.

## Method validation

Before testing with real users, validating each system component was essential. To this end, a series of simulations and controlled tests were carried out to evaluate the system’s reliability, responsiveness, and safety. The analysis of voltage signals, motor synchronization, and speed control demonstrated consistent and predictable behavior. These validations confirmed the system’s ability to translate head gestures into accurate movement commands with minimal delay and high reliability. Additionally, both the PWM and signal processing modules demonstrated stable performance across all tested configurations, with no signal loss or misinterpretation—highlighting the system’s readiness for real-time deployment.

**Fig. 9:**
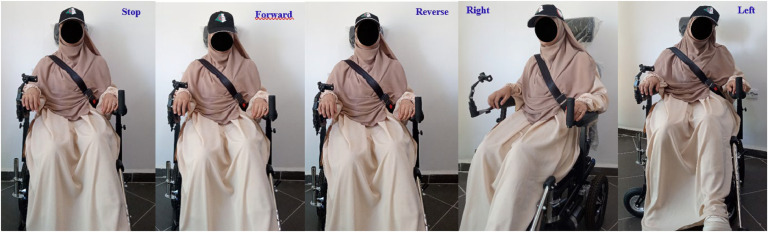
Head gesture control for wheelchair navigation: Visual feedback.

Fig. 9 showcases five images that vividly illustrate the actual movements used to control the wheelchair. These images capture how natural head gestures—tilting, turning, and nodding—are translated into precise commands. Each photo highlights a specific action, demonstrating the system’s ability to recognize even subtle shifts in head position. For instance, slight tilts forward or backward correspond to movement in those directions, while turning the head left or right steers the wheelchair accordingly.

This intuitive system ensures that users’ head gestures are captured with high precision, enabling smooth navigation even in tight spaces or open paths. As users move their heads naturally, the system continuously adjusts the wheelchair’s trajectory, allowing for fluid directional transitions.

The technology’s adaptability to natural behavior fosters seamless interaction, without requiring complex commands or physical effort. By responding in real-time, the system provides a control experience that closely mirrors users’ natural intentions, promoting autonomy and confidence.

Moreover, the feedback mechanism ensures prompt responses to inputs. Consistent reactions build user trust, ensuring comfort and security in daily use. These results demonstrate the system’s effectiveness in delivering both comfort and reliability while promoting a greater degree of independence.

These experimental results confirm the system’s ability to reliably detect and distinguish between predefined head gestures with minimal latency and high classification accuracy. The consistency across trials suggests that the threshold-based detection strategy is robust under controlled conditions. Furthermore, the signal processing pipeline was effective in filtering noise and maintaining responsiveness—critical features for assistive mobility applications. This performance validates the feasibility of the proposed method and its potential for real-time deployment.

In summary, this study demonstrates the viability of a head-movement-based control interface for electric wheelchairs, offering a practical and low-cost alternative to more complex EEG- or camera-based systems. Compared to existing solutions based on machine learning or computer vision (e.g., Emotiv, Tobii), our method benefits from simplicity, real-time response, and ease of integration. However, a current limitation is that tests were conducted with healthy subjects in controlled environments. Further studies involving target users in diverse real-life settings are needed. This aligns with prior findings (e.g., Pineau et *al.*, 2011), which also reported variations in real-world performance. Future work will focus on clinical validation and long-term usability to ensure robustness across different contexts.

## Limitations

Despite its promising results, the proposed methodology presents some limitations. First, the system has been tested in controlled conditions with a limited number of users, which may not fully represent the diversity of real-world scenarios. Second, the current implementation relies on fixed threshold angles for head movement detection, which may need adaptation across different users. Future iterations will integrate adaptive learning mechanisms to improve robustness and personalization.

## Conclusion

The proposed head-movement-based wheelchair control system, leveraging the MPU6050 accelerometer, represents a significant advancement in assistive mobility technology. Unlike many conceptual prototypes, our system has been successfully implemented and tested on a real electric wheelchair with an actual user, demonstrating high responsiveness, precision, and smooth operation. Experimental validation confirms that a 45° head tilt enables effective control for movement and stopping, eliminating the need for manual input and significantly enhancing autonomy for individuals with severe motor impairments, particularly tetraplegic users.

This work contributes to the development of more inclusive and intelligent assistive systems, and bridges the gap between low-cost design and high functional performance. It also provides a practical foundation for real-world deployment, particularly in low-resource settings.

Future developments will follow a multi-phase strategy. First, the system will be tested in long-term conditions with patients, in clinical and domestic settings, to assess its usability, safety, and robustness over time. Second, to address diverse user needs, we will integrate adaptive input modalities such as voice, gaze, or EEG signals, enabling personalized control schemes. Third, ergonomic improvements and energy efficiency will be explored to enhance comfort and autonomy. Finally, we plan to incorporate IoT capabilities for smart environment interaction and investigate commercial implementation pathways, aligning with current trends in inclusive health technologies.

## Ethics statements

This study involved human participants. Informed consent was obtained from all participants before conducting the experiments. The study was conducted in accordance with ethical guidelines to ensure participant safety, confidentiality, and compliance with relevant ethical standards.

## CRediT authorship contribution statement

**Abdelhakim Haddoun:** Supervision, Conceptualization, Methodology, Writing – original draft. **Dâlel Djabri:** Methodology, Software, Data curation, Formal analysis. **Mallak Saidani:** Methodology, Software, Data curation, Formal analysis. **Mohamed Benbouzid:** Methodology, Writing – review & editing.

## Declaration of competing interest

The authors declare that they have no known competing financial interests or personal relationships that could have appeared to influence the work reported in this paper.

## Data Availability

Data will be made available on request.

## Appendix Supplementary materials

Image, application 1
