# Supplementary material for: Development and evaluation of a head-controlled wheelchair system for users with severe motor impairments
Source: MethodsX. 2025 Jul 5;15:103485. doi: 10.1016/j.mex.2025.103485 (PMC12275118; doi:10.1016/j.mex.2025.103485)
Supplement: Supplementary file 1 [file mmc1.pdf]

Institut de Recherche Dupuy de Lôme
UMR CNRS 6027

Université de Bretagne Occidentale
Institut de Recherche Dupuy de Lôme
IUT de Brest – Rue de Kergoat
CS 93837 – F-29238 BREST Cedex 03, FRANCE

Brest, March 31, 2025.

Elsevier MethodsX

Paper title: Development and Evaluation of a Head-Controlled Wheelchair System for Users with Severe Motor Impairments

Authors: Abdelhakim Haddoun, Dâlel Djabri, Mallak Saidani, and Mohamed Benbouzid

CONSENT

I, Ms. Dâlel Djabri, co-author of the aforementioned paper and depicted in Figure 8, hereby give my full consent for the publication of the photos included in this figure.

Ms. Dâlel DJABRI
